# Health Benefits of Dairy Products’ Consumption—Consumer Point of View

**DOI:** 10.3390/foods13233925

**Published:** 2024-12-05

**Authors:** Dominika Jakubowska, Aneta Zofia Dąbrowska, Katarzyna Staniewska, Katarzyna Kiełczewska, Katarzyna E. Przybyłowicz, Justyna Żulewska, Adriana Łobacz

**Affiliations:** 1 Faculty of Economic Sciences, Department of Market and Consumption, University of Warmia and Mazury in Olsztyn, Oczapowskiego 2, 10-719 Olsztyn, Poland; 2 Faculty of Food Science, Department of Dairy Science and Quality Management, University of Warmia and Mazury in Olsztyn, Oczapowskiego 7, 10-719 Olsztyn, Poland; anetazj@uwm.edu.pl (A.Z.D.); kaka@uwm.edu.pl (K.K.); jzulewska@uwm.edu.pl (J.Ż.); adriana.lobacz@uwm.edu.pl (A.Ł.); 3 Faculty of Food Science, Department of Commodity Science and Food Analysis, University of Warmia and Mazury in Olsztyn, Pl. Cieszyński 1, 10-726 Olsztyn, Poland; kasta@uwm.edu.pl; 4 Faculty of Food Science, Department of Human Nutrition, University of Warmia and Mazury in Olsztyn, Słoneczna 45F, 10-718 Olsztyn, Poland; katarzyna.przybylowicz@uwm.edu.pl

**Keywords:** dairy products, consumer perceptions, health benefits, nutrition, consumer behaviour

## Abstract

The aim of this study was to identify and analyse consumer perceptions regarding the health benefits of different dairy products in Poland. This study examines the consumption frequency of selected dairy products in Poland and the health benefits which consumers associate with their regular consumption. It also explores how demographic factors, such as age, gender, and consumption frequency, influence these perceptions and identifies which dairy products are the most linked to specific health benefits. This study involved a quantitative survey of a representative sample of 2009 Polish consumers aged 19–30 and 66–75 years. This study revealed that the vast majority of the consumers recognise the health benefits of dairy products, particularly in areas such as better bone health, enhanced immune function, and improved digestion. The benefits associated with the regular consumption of milk, natural fresh cheeses, and natural fermented milk beverages were most frequently recognised. The respondents’ age had no significant effect on their perceptions of the health benefits of the selected dairy products. A statistically significant effect of gender was found only for the perceived benefits of consuming flavoured, fermented milk beverages. The frequency of consumption had a significant effect on the respondents’ perceptions of the benefits of all the studied groups of dairy products. In general, the consumers had positive attitudes towards the dairy products and believed in their potential health benefits. These findings have important implications for policy-makers. They underline the need for targeted public health campaigns to promote the consumption of dairy products as part of a balanced diet, emphasizing their specific health benefits. Such efforts could be especially effective if tailored to demographic factors such as gender and dietary habits.

## 1. Introduction

Consumer perceptions regarding the health benefits of food products have attracted considerable research interest in recent years, particularly in the context of dynamic economic and social changes that have intensified after the COVID-19 pandemic [1,2]. The pursuit of a healthy lifestyle and a balanced diet has increased the demand for food products that deliver health benefits and enhance consumers’ wellbeing. Healthy lifestyle promotion in mass media has also contributed to the popularity of functional foods, namely foods that offer health benefits beyond their nutritional value [3]. Dairy products play a particularly important role in health promotion in countries such as Poland, where the traditional diet is largely based on the regular consumption of milk, cheeses, and fermented milk beverages. The monthly per capita consumption of milk in Poland is three litres. The major dairy products consumed in Poland are milk, cheese, natural fresh cheese (tvorog), cream, yoghurt, and butter [4].

Dairy products are appreciated by consumers not only for their high nutritional value, but also for their health-promoting properties and desirable sensory attributes. Milk and dairy products are rich in the essential components important for healthy diet. Milk contains various bioactive nutrients that help maintain bodily functions. Protein is the most important and essential macronutrient required for virtually all life processes. It is a source of amino acids that act as the building blocks of tissues, especially in young organisms, and help regenerate tissue proteins in adults. Casein stimulates systemic immunity and plays a vital role in the transport of minerals, in particular calcium and phosphorus [5,6,7,8,9]. Whey proteins transport iron and have anti-inflammatory, antibacterial, antioxidant, anticarcinogenic, and immunomodulatory properties [7,9,10,11]. Casein and whey proteins are isolated from milk and used as functional ingredients in the production of various foods [6,10]. Milk proteins, including casein fractions and whey proteins, are a source of peptides that may have biological activity. Bioactive peptides help reduce hypertension; inhibit platelet aggregation; demonstrate opioid agonistic or antagonistic activity; transport minerals and trace elements; and exert immunostimulatory, antimicrobial, and antioxidant effects [7,11,12,13,14,15]. Milk fat is dispersed in the form of globules that are coated by a membrane composed of polar lipids (including phospholipids) and proteins, which preserves the integrity of milk fat globules. The phospholipids and proteins in the milk fat globule membrane (MFGM) are valuable functional and health-promoting components of milk and dairy products [9,16,17]. As components of cell membranes, polar lipids provide protection against the adverse effects of stress and bacterial and chemical damage to the gastric mucosa; exert neuroprotective effects; promote memory and concentration; and participate in the intracellular and intercellular signalling pathways [16,17,18]. Milk fat contains conjugated linoleic acid which exerts anti-atherosclerotic, antioxidant, and anticarcinogenic effects, prevents obesity and type two diabetes, and stimulates the immune system [19,20]. Milk fat is not a rich source of linoleic (v-6) or linolenic (v-3) acids, but it has a v-6:v-3 ratio of nearly one, which is considered optimal for human health [7,8].

Milk and dairy products are highly abundant in bioavailable calcium. Casein micelles contain colloidal calcium which promotes the absorption of casein phosphopeptides in dairy products that have been fermented, processed with the use of proteolytic enzymes, or formed during digestion. Milk has a favourable calcium-to-phosphorus ratio, and it contains lactose and vitamin D, which facilitate calcium absorption [21,22]. Calcium is essential for bone formation; it plays an important role in the prevention of osteoporosis, cardiovascular diseases, and type two diabetes, and contributes to weight loss [22]. Fat-soluble and water-soluble vitamins, which are present in milk and dairy products in different concentrations, exhibit a wide range of biological activities, play various roles in biochemical processes, and promote bodily functions [7,23,24]. Milk and dairy products play a very important role in the production of functional foods because they are characterised by a high content of bioactive compounds and optimal physiological ratios of nutrients. Some ingredients are removed and others, in particular individual bioactive compounds, are added in the process of designing health-promoting foods for specific groups of consumers. Care is also taken to preserve the naturally occurring ingredients that provide health benefits.

The evolving state of knowledge about the undoubted nutritional and health properties of milk and dairy products, confirmed by numerous scientific studies, may not always go hand in hand with the perception of dairy products and their market attributes according to consumers. The modern consumer perceptions of dairy products have evolved, influenced by trends in health awareness, sustainability, and dietary diversity [25]. On the global market, health benefits resulting from dairy product consumption are both a guarantee and a source of concern. On the one hand, the scientific evidence underscores the role of dairy in bone health, muscle development, and gut microbiota regulation through probiotics [25,26]. On the other hand, concerns over lactose intolerance, the environmental footprint of dairy farming, and the ethical treatment of animals have led to growing interest in plant-based alternatives [27]. Recent studies reveal that consumer choices are increasingly guided by a combination of health consciousness and ethical values. For instance, products like A2 milk, touted for its digestibility, and fermented dairy products enriched with probiotics are gaining traction among health-oriented consumers [25,26]. Simultaneously, functional dairy products infused with antioxidants and other bioactive compounds represent a growing niche that appeals to individuals seeking targeted health benefits [25,28].

Despite the well-documented benefits, the consumer perceptions of dairy products vary widely across different demographic groups, influenced by factors such as age, gender, and dietary habits. Age plays a pivotal role in shaping the attitudes towards dairy consumption. While younger consumers often focus on sensory experiences and cultural factors, older adults tend to value traditional dairy for its perceived nutritional benefits and familiarity [29]. Gender also influences consumption patterns, with women more likely to choose low-fat or functional dairy products, reflecting a focus on health and weight management [30]. Dietary habits further affect perceptions, as individuals adhering to vegetarian, vegan, or lactose-intolerant diets seek alternatives that align with their restrictions and values [31]. This indicates that the consumer preferences regarding the consumption of dairy products are evolving, which is not always solely due to the level of nutritional and health awareness [32]; therefore, the perception of the health benefits of milk and dairy products should be examined.

This study aims to fill a gap in the literature by examining how Polish consumers perceive the health benefits of various dairy products and to identify the products which, in the respondents’ opinion, provide the greatest health benefits. To achieve this, the research focuses on four key research questions:

1. What is consumption frequency of selected dairy products in Poland?
2. What are the perceived indications of health benefits resulting from regular consumption of selected dairy products?
3. Which dairy products, in the consumers’ opinion, are most frequently associated with specific health benefits?
4. How do consumer demographics, such as age, gender, and consumption frequency, influence the perceptions of the health benefits of dairy products?

The presented results can contribute to the literature on the topic by offering insights into how dairy products are perceived by consumers and what health benefits they expect to receive. The results offer practical guidance for policy-makers and industry stakeholders in promoting the consumption of dairy products as part of a balanced diet, providing a foundation for targeted public health campaigns. It also highlights the importance of consumption frequency in shaping perceptions of health benefits and provides new insights into the relationship between eating habits and perceived benefits.

## 2. Materials and Methods

### 2.1. Sample Collection

Polish consumers’ perceptions regarding the health benefits of dairy products were determined in a survey involving a representative, nationwide sample of 2009 consumers aged 19–30 and 66–75 years. The survey was conducted in January and February 2020 using the Computer-Assisted Personal Interview (CAPI) technique. A quota–random sample was selected for the survey. The sample was compiled based on the following population characteristics, the respondents’ place of residence, including voivodship and population in the place of residence (rural areas; cities with a population of up to 20,000; cities with a population of 20,000–50,000; cities with a population of 50,000–100,000; cities with a population of 100,000–200,000; cities with a population of 200,000–500,000; and cities with a population larger than 500,000); gender; and age in two age groups: 19–30 and 66–75 years. The ranges of age groups were established to compare the two different generations of consumers. Random sampling with replacement was used to select boroughs from each voivodship, and then quota sampling to select the respondents until the required and proportional number of citizens was collected in each voivodship according to The Polish Central Statistical Office. The application of such mixed sampling is allowed according to the available literature [33].

### 2.2. Questionnaire

A structured questionnaire was developed to evaluate consumer perceptions regarding the six main health benefits of consuming 11 types of dairy product. The selection of six main health benefits, such as weight management, heart health, bone health, digestive health, dental health, and immune defence, for inclusion in the questionnaire was based on the works of Korhonen [8] and Park [14], but the respondents could also indicate no health benefits. Thus, the consumers could indicate any number of the above-mentioned health benefits (including “no health benefits” as the seventh option) of consuming the following dairy products selected for the questionnaire according to the literature [34]:

1. Milk;
2. Milk powder (e.g., after reconstitution);
3. Condensed milk (including sweetened);
4. Sour cream and sweet cream;
5. Non-fermented milk beverages (such as cocoa milk and flavoured milk);
6. Natural fermented milk beverages (such as buttermilk, kefir, and yogurt);
7. Flavoured fermented milk beverages (such as fruit buttermilk, fruit kefir, and fruit yogurt);
8. Butter and butter-like products;
9. Natural fresh cheeses (such as homogenised cheese, fresh cheese spreads, tvorog, and cottage cheese—so called in Polish language as “tvorog cheeses”);
10. Flavoured fresh cheeses (such as fruit-, vanilla-, and vegetable-flavoured homogenised cheeses, fresh cheese spreads, tvorogs, and cottage cheeses);
11. Ripened cheeses (including so-called yellow cheeses and mould cheeses).

Milk desserts and ice cream were not included in this study. The respondents were also asked about gender, age group, and frequency of dairy product consumption.

### 2.3. Statistical Analysis

The following descriptive statistics were used in statistical analysis:

- Quartiles—to divide the dairy products into four groups based on the number of indicated health benefits;
- Median—to identify the most widely acknowledged health benefits for each of the 11 examined dairy products;
- Chi^2^ statistic at the significance level of 0.05 and Cramer’s V coefficient—to determine the influence of the respondents’ gender, age, and consumption frequency on the number of identified health benefits.

All the results were processed in Microsoft Excel 2010.

## 3. Results

The analysis of the frequency of dairy consumption revealed diverse consumption patterns (Table 1). The frequency of consumption was based on self-reported data.

Most respondents consumed milk several times per week, followed by once a day. Milk powder and condensed milk were the least frequently consumed dairy products. Sour cream and sweet cream were consumed frequently, usually several times per week. Non-fermented milk beverages were not consumed or were consumed several times per year by more than 40% of the respondents. In turn, most respondents consumed natural fermented milk beverages several times per week. Flavoured fermented milk beverages were slightly less popular, and these products were not consumed or were consumed several times per year by more than 25% of the respondents. A nearly identical number of the surveyed subjects consumed flavoured fermented milk beverages several times per week. Butter and butter-like products were most frequently consumed, usually several times a day. Most respondents consumed natural fresh cheese and flavoured fresh cheese several times per week. Ripened cheese was also most often consumed several times per week. Overall, most respondents consumed dairy products several times per week (6265 persons), whereas the smallest group of respondents consumed dairy products several times per day (1151 persons). A smaller number of respondents consumed dairy products once a day (2182 person). A substantial number of the surveyed subjects consumed dairy products 1–3 times per month (3405 persons) and once a week (3493 persons). Butter and butter-like products as well as milk were consumed most frequently (several times a day by 679 and 162 respondents, respectively), whereas milk powder and condensed milk were least frequently consumed (never or several times per year by 1413 and 1293 respondents, respectively). These results indicate that dairy products play an important role in Polish consumers’ diets.

**Figure 1:**
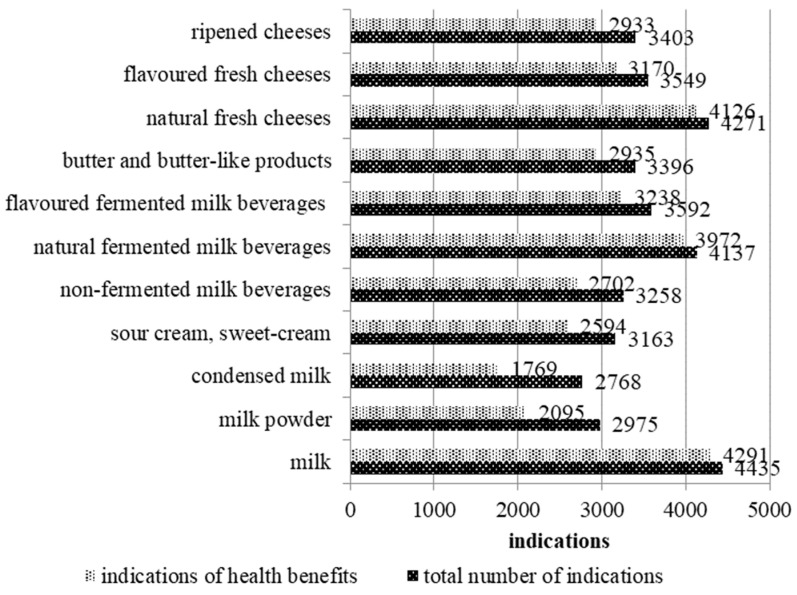
Total number of indications (n = 38,947, including indications of “no health benefits” option) and number of indications of health benefits resulting from regular consumption of 11 groups of dairy products (n = 33,825; Q3 = 3605; Q2 = 2935; Q1 = 2648).

The perceived health benefits associated with regular consumption of 11 groups of dairy products were examined in the next stage of analysis. A total of 38,947 responses were obtained in the question concerning the health benefits of regular consumption of 11 groups of dairy products (Figure 1). In 13% of the cases, the respondents were of the opinion that the regular consumption of selected dairy products does not provide any health benefits. Only 50 respondents did not associate any health benefits with any of the analysed dairy products. Most of them were young men.

Quartiles were computed to classify the 11 analysed groups of dairy products based on the number of health benefits identified by the respondents (n = 33,825; Q3 = 3605; Q2 = 2935; Q1 = 2648). Based on the number of indications, the dataset was divided into the following four groups:

1. Group 1—products with the highest number of perceived health benefits (equal or above Q3 = 3605): milk (4291 indications), natural fresh cheeses (4126), and natural fermented milk beverages (3972);
2. Group 2—products with a high number of perceived health benefits (equal or above Q2 = 2935): flavoured fermented milk beverages (3238), flavoured fresh cheeses (3170), and butter and butter-like products (2935);
3. Group 3—products with an average number of perceived health benefits (equal or above Q1 = 2648): ripened cheeses (2933) and non-fermented milk beverages (2702);
4. Group 4—products with a low number of perceived health benefits (equal or below Q1 = 2648): sour cream and sweet cream (2594), milk powder (2095), and condensed milk (1769).

Values exceeding the median (478) are marked in bold in Table 2. These values were used to identify the most widely acknowledged health benefits associated with each of the 11 analysed dairy products.

The dairy products with the highest number of perceived health benefits were milk (4435), natural fresh cheeses (4271), and natural fermented milk beverages (4137) (Table 2). Regular milk consumption was most frequently associated with healthy bones (1390 indications), followed by increased immunity (718 indications), healthy teeth (684 indications), a healthy heart (555 indications), and a healthy digestive tract (535 indications). The regular consumption of natural fresh cheeses was also most often associated with healthy bones (1108 indications), followed by increased immunity (732 indications), a healthy digestive tract (725 indications), healthy teeth (562 indications), and a healthy heart (533 indications). Therefore, the same health benefits were associated with milk and natural fresh cheeses, but they were ranked in a somewhat different order. According to the respondents, natural fermented milk beverages are most helpful in promoting healthy bones (921 indications), a healthy digestive tract (901 indications), and increased immunity (838 indications).

The number of above-median values decreased steadily in the subsequent product groups. Interestingly, bone health was regarded as the main benefit of regular dairy products’ consumption, whereas dairy products’ role in maintaining normal body weight was less often recognised.

The respondents’ opinions concerning the benefits of regular consumption of selected dairy products were influenced by gender, age, and consumption frequency.

The benefits of regular milk consumption were more often recognised by women than men (Table 3). Most female respondents were of the opinion that milk promotes healthy bones, increased immunity, and healthy teeth. Similar opinions were expressed by the men. The analysis of the age groups revealed that the health benefits of milk were recognised by a slightly higher number of younger (19–30 years) than older (66–75 years) respondents. The frequency of milk consumption influenced the respondents’ perceptions concerning the health benefits of this product. The benefits of milk were most frequently recognised by the persons who consumed milk several times per week and once a day. In turn, the respondents who did not consume milk or consumed milk several times per year were most often of the opinion that this dairy product does not deliver any health benefits. Overall, the health benefits associated with regular consumption of milk were more frequently identified by women and the respondents aged 19–30 years. These benefits were most frequently recognised by the respondents who consumed milk several times per week (1848 indications) or once a day (928 indications), whereas the persons who consumed milk rarely or not at all were more likely to indicate that milk does not provide any health benefits (59 indications).

The health benefits of natural fresh cheese were more often recognised by the female than the male respondents (Table 4). Most women were of the opinion that the consumption of natural fresh cheese promotes healthy bones, a healthy digestive tract, and increased immunity. The male respondents indicated that natural fresh cheese contributes to healthy bones, increased immunity, and a healthy digestive tract. Similar numbers of the male and female respondents were of the opinion that natural fresh cheese does not deliver health benefits. The analysis of the age groups revealed that the younger (19–30 years) respondents were only somewhat more likely to recognise the health benefits of natural fresh cheese than the older subjects (66–75 years). Most young respondents indicated that consumption of natural fresh cheese contributes to healthy bones, a healthy digestive tract, and increased immunity. These indications were similarly distributed in the group of older respondents. A higher number of younger than older respondents were of the opinion that natural fresh cheese does not provide health benefits. The persons consuming natural fresh cheese several times per week were most likely to indicate that this dairy product has health-promoting properties. In this group, the highest number of respondents were of the opinion that natural fresh cheese contributes to healthy bones, increased immunity, and a healthy digestive tract. The smallest number of health benefits was identified by the respondents who consumed natural fresh cheese several times per day. In the group of 200 subjects who consumed natural fresh cheese least frequently (never or several times per year), 42 respondents were of the opinion that this dairy product does not confer any health benefits. Overall, the health benefits associated with regular consumption of natural fresh cheese were more frequently identified by the women and the younger respondents. The respondents who consumed natural fresh cheese frequently were more likely to recognise its health benefits, whereas the persons who rarely consumed natural fresh cheese were more often of the opinion that this dairy product does not have health-promoting properties.

The respondents’ opinions concerning the health benefits of regular consumption of natural fermented milk beverages were also influenced by gender, age, and consumption frequency (Table 5). The health benefits conferred by natural fermented milk beverages were more often recognised by the women than the men. Most women were of the opinion that natural fermented milk beverages contribute to a healthy digestive tract, healthy bones, and increased immunity. The men expressed similar opinions. The number of respondents who indicated that fermented milks do not deliver any health benefits was similar in both the gender groups. The younger (19–30 years) and older (66–75 years) respondents had similar beliefs regarding the health benefits of regular consumption of natural fermented milk beverages. Maintaining a healthy digestive tract, healthy bones, and increased immunity were most frequently indicated in both the age groups. Healthy bones were most frequently indicated by the older respondents, whereas a healthy digestive tract was most often indicated by the younger respondents. The number of persons who did not identify any health benefits of fermented milk was slightly higher in the younger group than in the older group. The health-promoting properties of fermented milk were most often identified by the persons consuming these products several times per week and 1–3 times per month. In the group of 591 respondents who consumed these products least frequently (never or several times per year), 72 were of the opinion that fermented milk does not confer any health benefits. Overall, the women were more likely to recognise the health benefits of consuming natural fermented milk beverages. These benefits were recognised by both the younger and older consumers, but the respondents who consumed natural fermented milk beverages more frequently tended to have more positive opinions. The persons who rarely consumed natural fermented milk beverages were more likely to indicate that these products do not confer any health benefits.

The Chi^2^ test revealed that gender, age, and consumption frequency influenced the respondents’ perceptions regarding the health benefits of the 11 dairy products (Table 6). The null hypothesis (H_0_) stating that gender has no effect on the perceived health benefits was accepted for most of the examined dairy products. Flavoured fermented milk beverages with a Chi^2^_emp_ value of 13.14 were the only exception, and the null hypothesis was rejected in favour of the alternative hypothesis stating that gender influenced the respondents’ perceptions. The values of Cramer’s V were low for the remaining products, which additionally confirmed that gender had no significant effect on perceptions of health benefits. The respondents’ perceptions concerning the health-promoting properties of dairy products were not influenced by age; therefore, the null hypothesis was accepted. In contrast, consumption frequency significantly affected the respondents’ perceptions of the health benefits of all the analysed groups of dairy products; therefore, the null hypothesis was rejected in favour of the alternative hypothesis. The highest values of Chi^2^_emp_ were noted for condensed milk (368.69) and milk (350.91), which indicate that the consumption frequency significantly influenced the perceived health benefits of these products. The values of Cramer’s V were higher for consumption frequency than for the remaining independent variables, which points to a stronger association between consumption frequency and the perceived health benefits.

Overall, the respondents’ perceptions regarding the health benefits of dairy products were minimally affected by gender and age, excluding the flavoured fermented milk beverages, where gender played a role. In turn, consumption frequency significantly influenced the respondents’ perceptions of the health benefits conferred by all the analysed groups of dairy products.

## 4. Discussion

The present study was undertaken to determine consumer perceptions regarding the health benefits of milk and dairy products in a representative sample of Polish consumers aged 19–30 and 66–75 years. These products are relatively frequently, routinely, or even habitually chosen and purchased in Poland. However, it should be stressed that due to the steady improvement in the financial status of Polish consumers and the decreasing role of economic factors in their dietary choices, their purchasing decisions regarding various dairy products are increasingly influenced by psychological factors, which are more personal and subjective [35,36,37]. The research results confirm that the consumers take into account many attributes related to quality, i.e., safety, authenticity, naturalness, and healthiness [38]. According to Kurajdova et al. [39], milk and dairy products are consumed mainly because of their high nutritional value, positive impact on consumer health, and role in disease prevention.

Milk is regarded as a health-promoting product because it contains naturally occurring bioactive compounds. These include protein that is abundant in exogenous amino acids, milk fat with a unique composition and properties, lipophilic and hydrophilic vitamins, minerals, enzymes, and growth factors. Milk also provides health benefits because it contains easily digestible ingredients, such as bioactive peptides that are released after the gastrointestinal digestion of proteins, and milk fat that is dispersed in the form of globules surrounded by MFGM, whose components are susceptible to digestive enzymes [7,8,40]. When consumed daily in the right proportions as part of a balanced diet, dairy products deliver health benefits by supplying the body with essential nutrients [41].

The main area of analysis was connected with the differentiated attitudes of the consumers towards the product groups. In the current study, the Polish consumers from both the age groups were of the opinion that milk, natural fresh cheeses, and natural fermented milk beverages provide the highest number of health benefits. These observations have been confirmed in many experimental studies [41,42,43,44], which have shown that a healthy diet supplemented with fermented dairy products may be better than a diet that excludes these foods. In recent years, the interest in the production of natural fermented beverages has increased for scientific and commercial reasons [45]. Additionally, the functional food market has seen intense growth, especially for yogurts over the last few decades, due to the ease of including pre- and probiotics [46]. We discovered the same finding in our research; besides milk, also natural fermented milk beverages and natural fresh cheeses (particularly tvorogs) turned out to be the important products which consumption, according to the respondents’ indications, has a beneficial effect on the human body. The health benefits associated with fermented dairy products can be largely attributed to microbial activity, including the hydrolytic conversion of lactose into lactic acid and the hydrolytic cleavage of proteins into shorter fragments containing bioactive peptides [7,47,48]. Fermented milk, perceived as an integral part of the diet, also delivers health benefits because it contains probiotics. Probiotic foods have numerous health-promoting properties because they alleviate the symptoms of lactose intolerance, enhance digestion, reduce cholesterol levels, promote a healthy gut microbiome, exert immunomodulatory effects, prevent osteoporosis, atherosclerosis, cancer, obesity, diabetes, and metabolic syndrome; and promote cardiovascular, digestive, immune, and hormonal health [9,42,48,49,50,51,52]. The health benefits of milk, in particular fermented milk, can also be attributed to the presence of bioactive peptides that are released from milk proteins during enzymatic digestion or are present in dairy products. In cheese, bioactive peptides are derived mainly from casein, and in fermented dairy products, from casein and whey proteins [7,8,11,12,14,15,53]. Antimicrobial peptides, immunoglobulins, lactose derivatives, and lactoferrin promote digestive health [8,14]. The components that are essential for bone and dental health include bioavailable calcium, proteins, bioactive peptides, and casein phosphopeptides, which prevent dental carries and promote the remineralization of dental enamel, as well as short-chain fatty acids that are produced during fermentation and play an important role in bone remodelling and the prevention of osteoporosis [52,54,55]. Calcium, proteins, and bioactive peptides, in particular anti-atherosclerotic peptides derived from κ-casein, enhance cardiovascular health. In turn, whey proteins, especially lactoferrin, and immunomodulatory peptides inhibit microbial activity and stimulate immunity by activating the body’s defence mechanisms [8,14,54]. Short- and medium-chain fatty acids and monoacylglycerols formed by the enzymatic hydrolysis of milk triacylglycerols and sphingolipid metabolism products have antibacterial and antiviral properties [7,8].

Another area of analysis is related to the most common health benefits of dairy products from a consumer point of view. In the present study, most respondents identified healthy bones, followed by increased immunity and a healthy digestive tract, as the most common health benefits of dairy products. This is in accordance with the finding that consumers may attach lots of importance to ingredients they know well and are confident about their health effects [38]. As other studies show [56], increasing the level of certain minerals in dairy products, i.e., calcium, which is a valuable bone building block, may be perceived positively by consumers, as it is widely believed that the above-mentioned minerals have health benefits. Milk ingredients affect bodily functions, which is why they play an important role in the prevention of some diet-related diseases. It should also be noted that in many dairy products, these ingredients enter into synergistic interactions and produce a combined effect greater than the sum of their separate effects. The regular consumption of milk and dairy products contributes to a balanced diet that contains all the essential nutrients in the right proportions, which is a very important consideration in the production of functional foods and the prevention and treatment of some diseases. Sajdakowska et al. [57] conducted a study of Polish adult consumers to determine the significance of health-related factors in their choice of dairy products. In the cited study, most respondents agreed with the statements that dairy products are a rich source of protein and that fermented milk boosts immunity.

The next area of analysis was related to perceived benefits of the regular consumption of dairy products depending on the respondents’ gender and age. In the current study, the respondents’ perceptions regarding the health benefits of dairy products were not significantly affected by age. Both the younger (19–30 years) and older (66–75 years) consumers expressed similar opinions on the health-promoting properties of the dairy products. Gender had a minimal impact on the perceived health benefits associated with most of the analysed dairy products, excluding flavoured fermented milk beverages, where the respondents’ perceptions were significantly influenced by gender. The women were more likely to recognise the health benefits associated with regular consumption of the analysed groups of dairy products than the men. Similar observations have been made in other studies and in recent reports analysing trends in dairy consumption. Women are most likely to recognise the health-promoting properties of products enriched with additional ingredients and functional foods, and this observation could play an important role in communicating the health benefits of milk and dairy consumption. Women have a greater interest in products that improve wellbeing, promote weight loss, and improve bone and digestive health [58,59].

Another area of analysis was related to perceived benefits of the regular consumption of dairy products depending on consumption frequency. Numerous health benefits of regular consumption of dairy products were recognised by both the genders and both the age groups, but the respondents’ perceptions were influenced mostly by the frequency of dairy consumption. The persons consuming dairy products more often were more likely to indicate a higher number of health benefits. This is an important observation because according to the literature data, more aware and demanding consumers can contribute to numerous product innovations in the food market, including health-promoting ones [60].

In addition, other studies have shown that consumers with high self-control, including in terms of eating habits, are more likely to indicate intentions for healthy eating and health behaviours in the future. Some consumers with high self-control are more likely to present healthy intentions and reduce unhealthy intentions [61]. This is therefore the premise for building marketing communications with this more demanding target group.

Milk was characterised by the highest values of Chi^2^_emp_, which suggests that milk consumption frequency exerted the greatest influence on the respondents’ perceptions regarding the health-promoting properties of milk. These results are consistent with the concept of building consumer trust, increasing consumer acceptance of novel products, and communicating their health-promoting properties. Trust in specific products and neophobia strongly correlate with consumer acceptance of dairy products, in particular novel foods that are advertised as having health benefits [58,62].

The results of this study could also contribute to the effective promotion of the analysed groups of dairy products, which are relatively cheap; contain minimally processed ingredients; are free or relatively free of preservatives, synthetic colorants, and flavours; and are manufactured with the use of well-established, sustainable, and, in many cases, traditional technologies [57,63,64].

The large and representative sample and the quota–random sampling method are the main strengths of this study. However, the narrow age ranges (19–30 and 66–75 years) were a certain limitation that prevented the comprehensive assessment of the perceived benefits in the entire population. The choice of these age groups was dictated by the initial research design. Another limitation stemmed from the use of questionnaires, where the frequency of dairy consumption was declared by the respondents, but actual consumption was not measured. Despite the use of validated tools, memory errors and bias (overestimation or underestimation of the survey data) cannot be ruled out. In addition, this study involved only Polish consumers, and the findings cannot be directly extrapolated to other countries and cultures.

The results of this study have practical implications because they indicate that dairy producers and marketing experts should emphasise the health benefits delivered by the advertised products, in particular in the areas of bone health, immune health, and digestive health, to reach health-conscious consumers. The gender-based differences in the respondents’ perceptions may be useful in developing marketing strategies targeting specific consumer groups. The results can also be incorporated in educational and promotional campaigns aiming to increase dairy consumption as part of a balanced diet.

Future research should target other population groups, including younger consumers or respondents from other countries, to expand our understanding of the global differences in consumer perceptions of dairy products. The influence of external factors, such as mass media or nutrition trends, on the perceived healthiness of dairy products should also be investigated. It would also be interesting to determine physicians’ knowledge about the health-promoting properties of milk and dairy products and their role in the prevention and treatment of various diseases. These findings could be valuable for both the scientific community and the dairy industry.

## 5. Conclusions

The results of this study, analysing Polish consumers’ perceptions regarding the health benefits of consuming milk and dairy products, indicated that milk, natural fresh cheeses, and natural fermented milk beverages were regarded as the dairy products that provide the highest number of health benefits. The respondents also recognised the health-promoting properties of flavoured fermented milk beverages and flavoured fresh cheeses. Ripened cheeses, butter and butter-like products, and non-fermented milk beverages were regarded as the dairy products that deliver moderate health benefits. Sour cream and sweet cream, milk powder, and condensed milk were perceived as the products with the lowest number of health benefits.

The most frequently recognised health benefits of dairy products were healthy bones, followed by increased immunity, and a healthy digestive tract.

Gender had a minimal impact on the perceived health benefits associated with most of the analysed dairy products, excluding flavoured fermented milk beverages, where the respondents’ perceptions were significantly influenced by gender. The women were more likely to recognise the health benefits associated with regular consumption of the analysed groups of dairy products than the men.

The respondents’ perceptions regarding the health benefits of dairy products did not differ significantly between the age groups. Both the younger (19–30 years) and older (66–75 years) consumers indicated that dairy products have health-promoting properties.

The frequency of dairy consumption exerted a significant influence on the respondents’ perceptions. The persons consuming dairy products more often were more likely to indicate a higher number of health benefits. Condensed milk and milk were characterised by the highest values of Chi^2^_emp,_ which suggests that consumption frequency exerted the greatest influence on the respondents’ perceptions regarding the health-promoting properties of these dairy products.

Both the women and men and the respondents from both the age groups recognised that regular dairy consumption confers numerous health benefits by promoting a healthy body weight, a healthy heart, healthy bones, increased immunity, a healthy digestive tract, and healthy teeth. However, the respondents’ perceptions were influenced mostly by the frequency of dairy consumption.

In summary, consumption frequency was related to the respondents’ perceptions concerning the health-promoting properties of dairy products. Gender and age had a smaller, but in some cases significant, impact on consumer perceptions.

These findings are relevant for public health campaigns and policy-makers, as they highlight the importance of promoting regular dairy consumption to enhance awareness of its health benefits. Tailored strategies could target demographic groups with lower consumption frequencies to encourage healthier dietary habits and improve the nutritional outcomes.

## Figures and Tables

**Table 1:** Consumption frequency of 11 groups of dairy products in Poland.

Product Group	Never or Several Times per Year		1–3 Times per Month		Once a Week		Several Times per Week		Once a Day		Several Times per Day	
	n	%	n	%	n	%	n	%	n	%	n	%
Milk	187	9.3	209	10.4	263	13.1	794	39.5	394	19.6	162	8.1
Milk powder	1413	70.3	161	8.0	119	5.9	200	10.0	93	4.6	23	1.1
Condensed milk	1293	64.4	253	12.6	139	6.9	216	10.8	80	4.0	28	1.4
Sour cream and sweet cream	196	9.8	342	17.0	441	22.0	812	40.4	185	9.2	33	1.6
Non-fermented milk beverages	818	40.7	446	22.2	257	12.8	349	17.4	110	5.5	29	1.4
Natural fermented milk beverages	346	17.2	490	24.4	410	20.4	586	29.2	144	7.2	33	1.6
Flavoured fermented milk beverages	544	27.1	414	20.6	368	18.3	529	26.3	125	6.2	29	1.4
Butter and butter-like products	92	4.6	84	4.2	146	7.3	487	24.2	521	25.9	679	33.8
Natural fresh cheeses	137	6.8	316	15.7	447	22.2	847	42.2	222	11.1	40	2.0
Flavoured fresh cheeses	373	18.6	393	19.6	450	22.4	630	31.4	135	6.7	28	1.4
Ripened cheeses	204	10.2	297	14.8	453	22.5	815	40.6	173	8.6	67	3.3
Total	5603	25.4	3405	15.4	3493	15.8	6265	28.3	2182	9.9	1151	5.2

n—number of indications.

**Table 2:** Perceived health benefits of regular consumption of 11 groups of dairy products.

Product Group	Benefits Associated with Regular Consumption of Dairy Products															
	Normal Body Weight		Healthy Heart		Healthy Bones		Increased Immunity		Healthy Digestive Tract		Healthy Teeth		No Health Benefits		Total	
	n	%	n	%	n	%	n	%	n	%	n	%	n	%	n	%
**Group 1—products with the highest number of perceived health benefits**																
Milk	409	9.2	**555**	12.5	**1390**	31.3	**718**	16.2	**535**	12.1	**684**	15.4	144	3.2	4435	100
Natural fresh cheeses	466	10.9	**533**	12.5	**1108**	25.9	**732**	17.1	**725**	17.0	**562**	13.2	145	3.4	4271	100
Natural fermented milk beverages	402	9.7	465	11.2	**921**	22.3	**838**	20.3	**901**	21.8	445	10.8	165	4.0	4137	100
**Group 2—products with a high number of perceived health benefits**																
Flavoured fermented milk beverages	287	8.0	403	11.2	**792**	22.0	**700**	19.5	**738**	20.5	318	8.9	354	9.9	3592	100
Flavoured fresh cheeses	323	9.1	415	11.7	**886**	25.0	**620**	17.5	**559**	15.8	367	10.3	379	10.7	3549	100
Butter and butter-like products	264	7.8	**578**	17.0	**664**	19.6	**636**	18.7	**496**	14.6	297	8.7	461	13.6	3396	100
**Group 3—products with an average number of perceived health benefits**																
Ripened cheeses	290	8.5	354	10.4	**794**	23.3	**604**	17.7	**545**	16.0	346	10.2	470	13.8	3403	100
Non-fermented milk beverages	239	7.3	366	11.2	**787**	24.2	**575**	17.6	427	13.1	308	9.5	556	17.1	3258	100
**Group 4—products with a low number of perceived health benefits**																
Sour cream and sweet cream	195	6.2	306	9.7	**786**	24.8	**489**	15.5	**491**	15.5	327	10.3	569	18.0	3163	100
Milk powder	189	6.4	260	8.7	**621**	20.9	440	14.8	293	9.8	292	9.8	880	29.6	2975	100
Condensed milk	157	5.7	231	8.3	**541**	19.5	353	12.8	267	9.6	220	7.9	999	36.1	2768	100

n—number of indications.

**Table 3:** Perceived benefits of regular consumption of milk depending on the respondents’ gender, age, and consumption frequency.

Independent Variables	Benefits Associated with Regular Milk Consumption														Total	
	Normal Body Weight		Healthy Heart		Healthy Bones		Increased Immunity		Healthy Digestive Tract		Healthy Teeth		No Health Benefits			
**Gender**	**n**	**%**	**n**	**%**	**n**	**%**	**n**	**%**	**n**	**%**	**n**	**%**	**n**	**%**	**n**	**%**
Female	229	56.0	294	53.0	735	52.9	379	52.8	296	55.3	377	55.1	74	51.4	2384	53.8
Male	180	44.0	261	47.0	655	47.1	339	47.2	239	44.7	307	44.9	70	48.6	2051	46.2
Total	409	100	555	100	1390	100	718	100	535	100	684	100	144	100	4435	100
**Age**	**n**	**%**	**n**	**%**	**n**	**%**	**n**	**%**	**n**	**%**	**n**	**%**	**n**	**%**	**n**	**%**
19–30 years	211	51.6	280	50.5	701	50.4	371	51.7	264	49.3	364	53.2	78	54.2	2269	51.2
66–75 years	198	48.4	275	49.5	689	49.6	347	48.3	271	50.7	320	46.8	66	45.8	2166	48.8
Total	409	100	555	100	1390	100	718	100	535	100	684	100	144	100	4435	100
**Frequency of consumption**	**n**	**%**	**n**	**%**	**n**	**%**	**n**	**%**	**n**	**%**	**n**	**%**	**n**	**%**	**n**	**%**
Never or several times per year	18	4.4	17	3.1	87	6.3	35	4.9	20	3.7	38	5.6	59	41.0	274	6.2
1–3 times per month	44	10.8	49	8.8	144	10.4	75	10.4	60	11.2	70	10.2	16	11.1	458	10.3
Once a week	51	12.5	57	10.3	173	12.4	95	13.2	58	10.8	73	10.7	16	11.1	523	11.8
Several times per week	156	38.1	246	44.3	601	43.2	302	42.1	216	40.4	294	43.0	33	22.9	1848	41.7
Once a day	87	21.3	130	23.4	269	19.4	146	20.3	127	23.7	152	22.2	17	11.8	928	20.9
Several times per day	53	13.0	56	10.1	116	8.3	65	9.1	54	10.1	57	8.3	3	2.1	404	9.1
Total	409	100	555	100	1390	100	718	100	535	100	684	100	144	100	4435	100

n—number of indications.

**Table 4:** Perceived benefits of regular consumption of natural fresh cheeses depending on respondents’ gender, age, and consumption frequency.

Independent Variables	Benefits Associated with Regular Consumption of Natural Fresh Cheeses														Total	
	Normal Body Weight		Healthy Heart		Healthy Bones		Increased Immunity		Healthy Digestive Tract		Healthy Teeth		No Health Benefits			
**Gender**	**n**	**%**	**n**	**%**	**n**	**%**	**n**	**%**	**n**	**%**	**n**	**%**	**n**	**%**	**n**	**%**
Female	261	56.0	284	53.3	598	54.0	394	53.8	399	55.0	312	55.5	72	49.7	2320	54.3
Male	205	44.0	249	46.7	510	46.0	338	46.2	326	45.0	250	44.5	73	50.3	1951	45.7
Total	466	100	533	100	1108	100	732	100	725	100	562	100	145	100	4271	100
**Age**	**n**	**%**	**n**	**%**	**n**	**%**	**n**	**%**	**n**	**%**	**n**	**%**	**n**	**%**	**n**	**%**
19–30 years	241	51.7	260	48.8	526	47.5	357	48.8	362	49.9	275	48.9	86	59.3	2107	49.3
66–75 years	225	48.3	273	51.2	582	52.5	375	51.2	363	50.1	287	51.1	59	40.7	2164	50.7
Total	466	100	533	100	1108	100	732	100	725	100	562	100	145	100	4271	100
**Frequency of consumption**	**n**	**%**	**n**	**%**	**n**	**%**	**n**	**%**	**n**	**%**	**n**	**%**	**n**	**%**	**n**	**%**
Never or several times per year	13	2.8	14	2.6	47	4.2	36	4.9	28	3.9	20	3.6	42	29.0	200	4.7
1–3 times per month	69	14.8	72	13.5	152	13.7	101	13.8	115	15.9	63	11.2	20	13.8	592	13.9
Once a week	92	19.7	115	21.6	273	24.6	165	22.5	152	21.0	119	21.2	26	17.9	942	22.1
Several times per week	221	47.4	231	43.3	496	44.8	331	45.2	323	44.6	283	50.4	40	27.6	1925	45.1
Once a day	54	11.6	85	15.9	118	10.6	79	10.8	92	12.7	65	11.6	15	10.3	508	11.9
Several times per day	17	3.6	16	3.0	22	2.0	20	2.7	15	2.1	12	2.1	2	1.4	104	2.4
Total	466	100	533	100	1108	100	732	100	725	100	562	100	145	100	4271	100

n—number of indications.

**Table 5:** Perceived benefits of regular consumption of natural fermented milk beverages depending on respondents’ gender, age, and consumption frequency.

Independent Variables	Benefits Associated with Regular Consumption of Natural Fermented Milk Beverages														Total	
	Normal Body Weight		Healthy Heart		Healthy Bones		Increased Immunity		Healthy Digestive Tract		Healthy Teeth		No Health Benefits			
**Gender**	**n**	**%**	**n**	**%**	**n**	**%**	**n**	**%**	**n**	**%**	**n**	**%**	**n**	**%**	**n**	**%**
Female	223	55.5	240	51.6	484	52.6	481	57.4	513	56.9	236	53.0	82	49.7	2259	54.6
Male	179	44.5	225	48.4	437	47.4	357	42.6	388	43.1	209	47.0	83	50.3	1878	45.4
Total	402	100	465	100	921	100	838	100	901	100	445	100	165	100	4137	100
**Age**	**n**	**%**	**n**	**%**	**n**	**%**	**n**	**%**	**n**	**%**	**n**	**%**	**n**	**%**	**n**	**%**
19–30 years	209	52.0	227	48.8	442	48.0	408	48.7	455	50.5	224	50.3	90	54.5	2055	49.7
66–75 years	193	48.0	238	51.2	479	52.0	430	51.3	446	49.5	221	49.7	75	45.5	2082	50.3
Total	402	100	465	100	921	100	838	100	901	100	445	100	165	100	4137	100
**Frequency of consumption**	**n**	**%**	**n**	**%**	**n**	**%**	**n**	**%**	**n**	**%**	**n**	**%**	**n**	**%**	**n**	**%**
Never or several times per year	56	13.9	46	9.9	126	13.7	98	11.7	130	14.4	63	14.2	72	43.6	591	14.3
1–3 times per month	98	24.4	114	24.5	219	23.8	216	25.8	241	26.7	97	21.8	26	15.8	1011	24.4
Once a week	85	21.1	100	21.5	221	24.0	181	21.6	202	22.4	106	23.8	21	12.7	916	22.1
Several times per week	124	30.8	154	33.1	282	30.6	278	33.2	260	28.9	145	32.6	36	21.8	1279	30.9
Once a day	28	7.0	42	9.0	64	6.9	51	6.1	53	5.9	24	5.4	9	5.5	271	6.6
Several times per day	11	2.7	9	1.9	9	1.0	14	1.7	15	1.7	10	2.2	1	0.6	69	1.7
Total	402	100	465	100	921	100	838	100	901	100	445	100	165	100	4137	100

n—number of indications.

**Table 6:** The results of the Chi^2^ test analysing the effects of the respondents’ gender, age, and consumption frequency on the number of the recognised health benefits of dairy products.

Product Group	Gender			Age			Frequency of Consumption		
	Chi^2^_emp_	Accepted Hypothesis	V-Cramer Coef.	Chi^2^_emp_	Accepted Hypothesis	V-Cramer Coef.	Chi^2^_emp_	Accepted Hypothesis	V-CramerCoef.
Milk	3.03	H_0_	0.026	2.90	H_0_	0.026	350.91	H_1_	0.126
Milk powder	11.96	H_0_	0.063	1.48	H_0_	0.022	265.23	H_1_	0.134
Condensed milk	1.67	H_0_	0.025	2.22	H_0_	0.028	368.69	H_1_	0.163
Sour cream and sweet cream	7.01	H_0_	0.047	2.98	H_0_	0.031	246.83	H_1_	0.125
Non-fermented milk beverages	5.66	H_0_	0.042	6.19	H_0_	0.044	280.55	H_1_	0.131
Natural fermented milk beverages	10.03	H_0_	0.049	4.26	H_0_	0.032	150.71	H_1_	0.085
Flavoured fermented milk beverages	13.14	H_1_	0.060	6.17	H_0_	0.041	298.48	H_1_	0.129
Butter and butter-like products	4.60	H_0_	0.037	10.66	H_0_	0.056	109.08	H_1_	0.080
Natural fresh cheeses	2.64	H_0_	0.025	8.67	H_0_	0.045	236.31	H_1_	0.105
Flavoured fresh cheeses	3.45	H_0_	0.031	2.76	H_0_	0.028	269.21	H_1_	0.123
Ripened cheeses	3.89	H_0_	0.034	3.08	H_0_	0.030	153.61	H_1_	0.095

where: H_0_—null hypothesis; H_1_—alternative hypothesis; Chi^2^_emp_—calculated Chi^2^; tabulated Chi^2^ = 12.57 for gender and age; df = 6 for gender and age; tabulated Chi^2^ = 43.77 for frequency of consumption; df = 30 for frequency of consumption; level of significance α = 0.05.

## Data Availability

Due to ethical restrictions and participant confidentiality, the data cannot be made publicly available. However, the data from this study are available upon request, for researchers who meet the criteria for access to confidential data. Data requests can be sent to the study coordinator (Katarzyna Eufemia Przybyłowicz).
